# Physiotherapy Management of an Adolescent With Pityriasis Rubra Pilaris Along With Rickets

**DOI:** 10.7759/cureus.30518

**Published:** 2022-10-20

**Authors:** Sakshi Palkrit, Neha Chitale, Pratik Phansopkar, Mitushi Deshmukh

**Affiliations:** 1 Department of Physical Therapy, Ravi Nair Physiotherapy College, Datta Meghe Institute of Medical Sciences, Wardha, IND; 2 Department of Musculoskeletal Physiotherapy, Ravi Nair Physiotherapy College, Datta Meghe Institute of Medical Sciences, Wardha, IND

**Keywords:** gait training, physiotherapy, physiotherapy management, pityriasis rubra pilaris, rickets

## Abstract

Rickets is an ossification and mineralization disorder of the growth plate before skeletal maturity that is peculiar to children and adolescents. Most children are affected by this deficient disorder throughout their skeletal growth stage, characterized by deformed and soft bones, due to a failure to assimilate and utilize calcium and phosphorus properly. It is most frequent in children aged four months to three years in developing countries. In the Indian subcontinent, it continues to be a major health problem. The majority of rickets symptoms include bone pain, deformity of the bones, and impaired growth velocity. In addition to damaging the skeletal system, it also affects other systems, which results in substantial morbidity. The term "rachitic pneumopathy" has long been used to describe respiratory issues caused by rickets. Pityriasis rubra pilaris (PRP) is an inflammatory rare skin disease that affects children as well as adults of all ages. They can develop PRP's clinical characteristics and individual prognoses are quite varied. Rickets occurring in association with skin diseases is rare. Here, we report an instance of a 15-year-old boy who gave a history of pain and swelling in the knees for the past three months. He couldn’t bear weight and had walking difficulty as well. Along with this, he also complained of itchy lesions for nine years. Initially, the lesions were on the right arm, which progressed to the left arm, chest, back and abdomen. Later, they were seen on the lower limbs. After appropriate diagnostic work, he was diagnosed with rickets with pityriasis rubra pilaris. The patient received both medical and physical therapy treatment. The physical therapy rehabilitation program used in this case study significantly improved the patient's functional independence by reducing pain and improving joint mobility, muscle strength, endurance, and gait. After six weeks of rehabilitation, there was an improvement in the ranges of the joint, strength of muscle, gait, and functional independence significantly using physical therapy techniques. This case study shows the value of comprehensive physical therapy in a case of pityriasis rubra pilaris with rickets in a 15-year-old boy.

## Introduction

Rickets is an ossification and mineralization disorder of the growth plate before skeletal maturity that is unique to children and adolescents [1]. The majority of children are affected by this deficient disorder throughout their skeletal growth stage, characterized by deformed and soft bones; it is caused due to a failure to properly assimilate and utilize calcium and phosphorus [2]. It is most frequent in children aged four months to three years in developing countries. However, kids of vitamin D-deficient mothers have been known to develop congenital rickets and rickets in the early months of life [3]. Solar vitamin D insufficiency and/or dietary calcium inadequacy are the major causes of rickets globally. Rickets may have severe health effects that go beyond bone abnormalities, such as life-threatening hypocalcemic seizures and, in infants, heart failure secondary to dilated cardiomyopathy [4]. Rickets is a metabolic bone disease that manifests itself in several ways such as a failure or delay to calcify the cartilaginous growth plate which has not yet fused its epiphyses in children [5]. The inability of endochondral ossification in growing bone is evidence of rickets [6]. Clinical presentation in infants born to mothers who are deficient, poor feeding, irritability, and hypocalcemic seizures are common in the neonatal period. Apnoea and stridor are rare symptoms. A soft skull (craniotabes) and big fontanelle may be found during an examination. Older infants may have developmental delays, hypocalcemic seizures, or, in rare cases, cardiomyopathy-related heart failure. Hypotonia, swollen joints (wrist, ankle, and costochondral junctions of the rib, also known as rachitic rosary), and heart failure symptoms are all clinical characteristics. Proximal muscular weakness, delayed development, aberrant dentition and fractures, swelling of wrists and ankles, rachitic rosary, leg bowing deformities, and stunting may be seen in children [4].

Pityriasis rubra pilaris (PRP) is a papulosquamous rare inflammatory dermatosis that is idiopathic in nature [7,8]. About one in 50,000 Indian individuals have this condition, and one in 500 new patients reported having a dermatologic condition in the pediatric population, which is a higher prevalence affecting all races and both sexes equally [7]. In order to categorize PRP, Griffiths created a system dividing it into six categories. Type I is the classical PRP for adults. Type II is atypical adult onset differing from type-I based on prolonged duration and characteristics of atypical morphology which includes a lamellar scale of the palms and soles, ichthyosiform scale, and occasionally partial alopecia. PRP types III-V are those with juvenile onset. The most prevalent variety of PRP in children is Type IV, which has a juvenile-circumscribed onset. Like Type II, Type V is an atypical juvenile PRP with ichthyosiform characteristics that is chronic. Patients may see sclerodermatous alterations to their fingers. According to Miralles et al., PRP brought on by HIV infection should be classified as type VI PRP, which is marked by poor prognosis, refractoriness to treatment, and nodulocystic and lichen spinulosis-like lesions [9]. Classic PRP in adults and children results in generalized body redness with islands of sparing which begins from the head and spreads downwards. The classic types usually disappear after three to five years. Atypical adult and juvenile PRP have a more pronounced red, dry rash that often affects the lower limbs and can persist for many years. Early infancy may be seen in atypical juvenile PRP, which may be inherited. The knees and elbows of circumscribed juvenile PRP have red, scaly patches. In people with HIV, PRP can occur in rare cases. A condition known as palmoplantar keratoderma affects the palms and soles of the majority of patients. This may make it difficult to walk and result in painful skin cracks. Hair loss, pain in joints, sensitivity to the sun, and itching can also occur. Ectropion, an eye disorder that can cause dryness, discomfort, and difficulties in seeing, may also occur [10]. Through this case report, the rationale was to provide insight into the role of a physiotherapist in pityriasis rubra pilaris along with rickets in a 15-year-old boy.

## Case presentation

Here is an instance of a 15-year-old boy, who came with his parents, and gave a history of pain and swelling in the knees for the past three months. Pain rating was 8/10 on the Numeric Pain Rating Scale (NPRS). It was gradually increasing in nature. He couldn’t bear weight and had walking difficulty as well. He was advised for an X-Ray and laboratory investigations like serum calcium, serum phosphorus, and alkaline phosphatase levels and was diagnosed with rickets.

**Figure 1:**
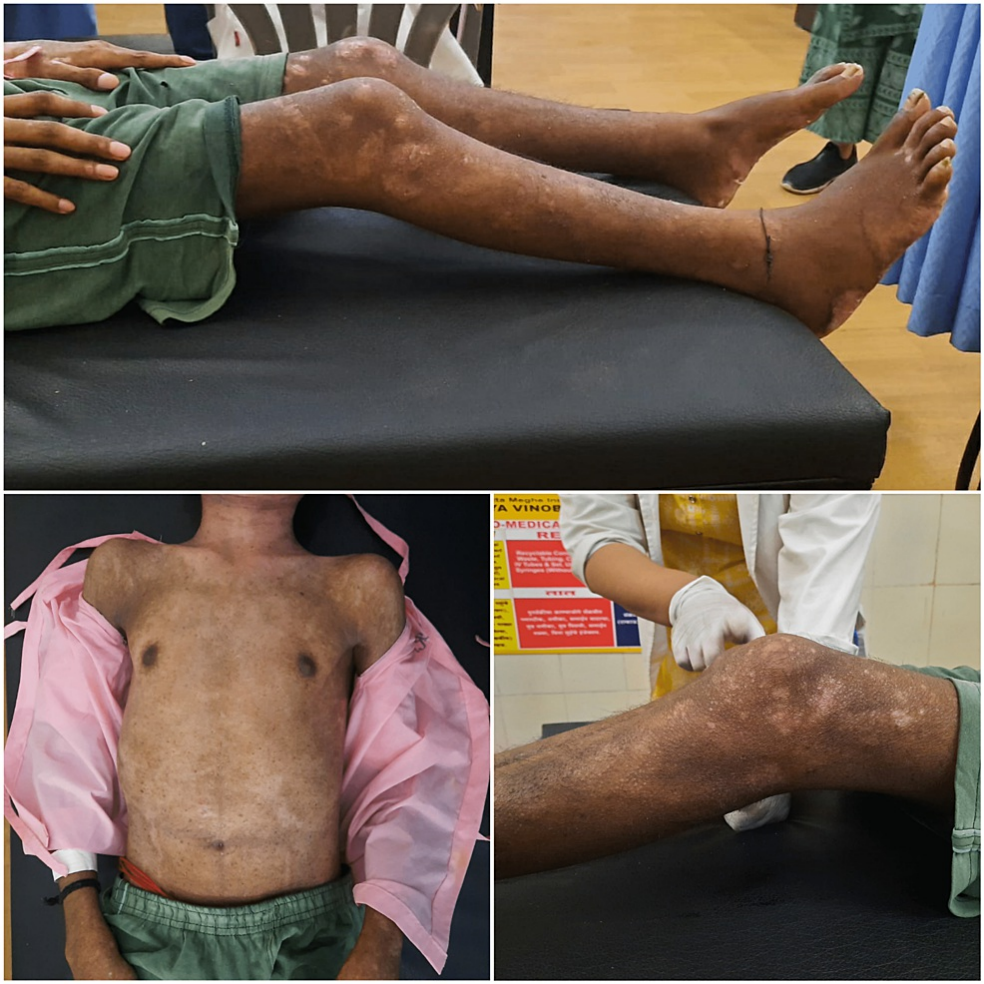
Lesions seen in the pityriasis rubra pilaris

Apart from this, the patient complained of itchy lesions for nine years. Initially, the lesions were on the right arm which progressed to the left arm, chest, back, and abdomen (Figure 1). Later, lesions on the lower limbs were seen which were progressive in nature. He also complained of aggravation of the lesions on exposure to sunlight. He took treatment for the skin lesions nine years back but no improvement in the symptoms was seen. For the past year, he had also complained of dryness all over the body. With all these complaints he was admitted to the hospital.

Following consultation, the requisite investigations were carried out; he was diagnosed with pityriasis rubra pilaris. Physiotherapy was recommended to the patient's family for further management of rickets.

Clinical findings

**Figure 2:**
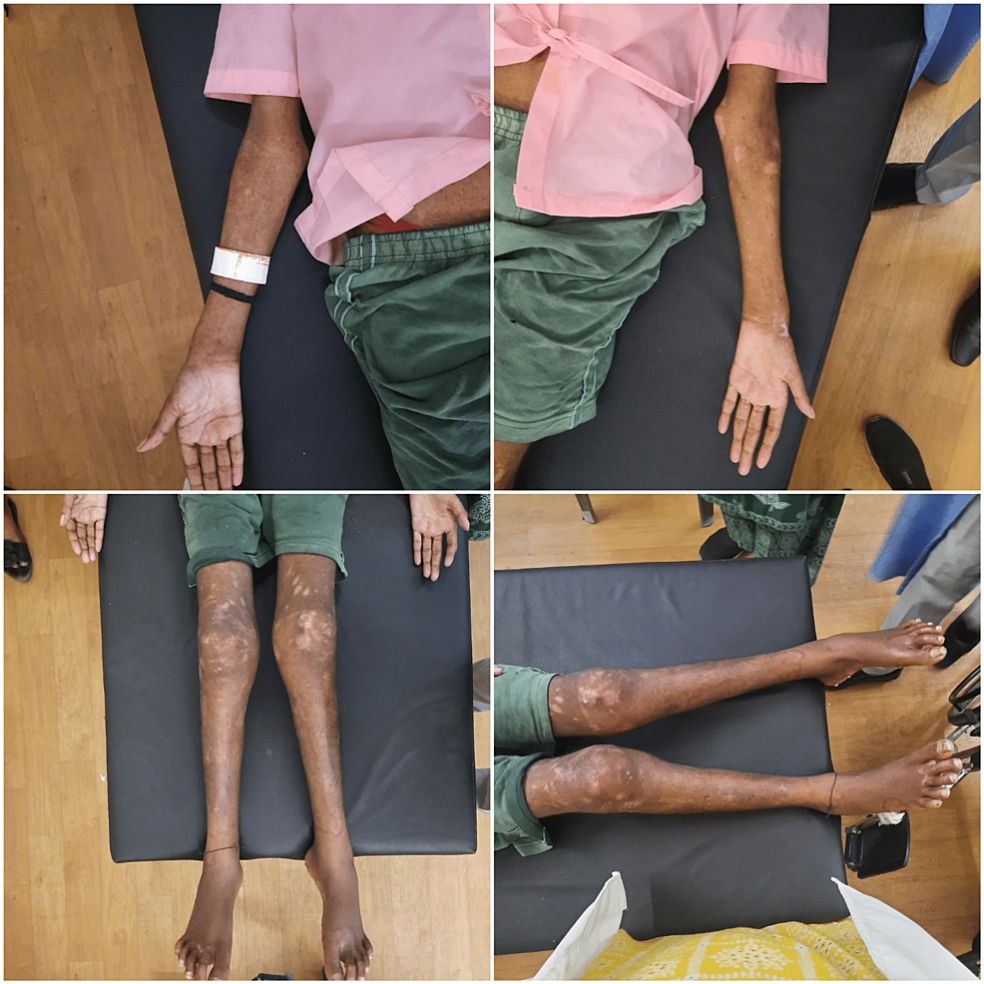
Shows widening of the elbow, genu valgum

The patient was informed about the physical examination and the investigations after obtaining written consent. While assessing, he was conscious and well-oriented about the time, place, and person. His assessment was in the supine position. On observation, the patient was in a supine lying position with an ectomorphic body build. Both shoulders were not on the same level. Widened elbows along with widening of the wrist joint bilaterally were observed (Figure 2). When examined, a barrel-shaped chest was observed along with a rachitic rosary present over the left costochondral area was observed. While examining the lower limbs, genu valgum was seen (Figure 2). Flexion deformity was observed in both knees. When the patient is in a sitting position a mildly protruded abdomen was seen. The range of motion was taken using a goniometer, which was reduced bilaterally. Manual muscle test (MMT) showed 2/5 for the shoulder flexors, elbow flexors, shoulder abductors and adductors, hip flexors and extensors, knee flexors, and extensors. For shoulder extensors, and elbow extensors the MMT was 3/5.

Diagnostic assessment

**Figure 3:**
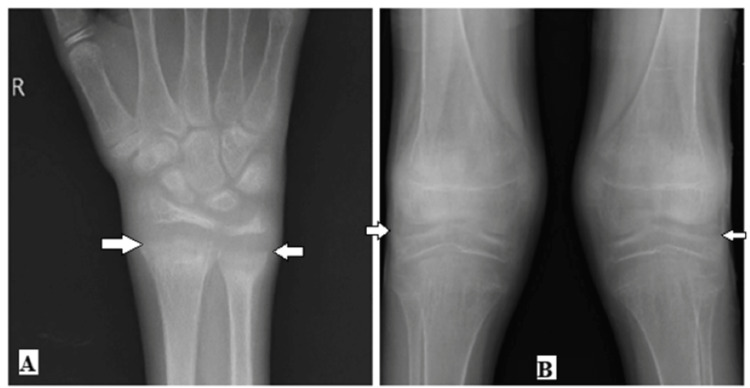
A- Shows fraying and cupping at the radial and ulnar. B- Shows the widening of the distal end of the knee joint

Laboratory investigations showed serum calcium and serum phosphorus were reduced and alkaline phosphatase levels were increased. X-ray of wrist showed cupping and fraying at the metaphyseal end in the wrist joint; X-ray of knees showed widening at the knee joint (Figure 3).

Therapeutic intervention

The rehabilitation programme was planned for 4-6 weeks and further home exercise programmes were prescribed with regular follow-ups. The maximum protection phase (day 1-day 7) is described in Table 1, the moderate protection phase (week 2-week 4) is described in Table 2, and the minimum protection phase (week 5-week 6) is described in Table 3.

**Table 1:** Maximum protection phase B/L = bilateral

Intervention	Dosage	Rationale
Interferential therapy (IFT)	10 minutes to B/L knee	To alleviate pain
Shoulder pulley, and wheel	Ten repetitions of one set	To enhance the joint mobility of the shoulder joint
Heel slides in supine	A set of ten repetitions	To enhance the mobility of knee joint
Active assisted range of motion exercises to the elbow, wrist, hip, and knee joint	Ten repetitions of a set	To maintain and enhance the joint mobility

**Table 2:** Moderate protection phase B/L = bilateral

Intervention	Dosage	Rationale
Interferential therapy	10 minutes to B/L knee	To alleviate pain
Shoulder, elbow, wrist, hip, and knee joint – active range of motion exercises	10 reps of one set	To maintain joint mobility
Heel slides in supine	A set of ten repetitions	To enhance the mobility of knee joint
Isometrics to shoulder, elbow, quadriceps, hamstrings, and vastus medialis oblique (VMO)	Ten reps of a set with ten seconds of hold	To strengthen the muscles
Cervical isometrics	10 reps with 10 seconds of hold	To strengthen cervical muscles
Static abdominals	Ten reps of a set with ten seconds of hold	To strengthen the muscles
Pelvic bridging	A set of ten reps	To build strength in hip extensors
Sit-to-stand activity	A set of ten reps	To make the patient stand
One leg stance with eyes closed with the support	Ten seconds hold with five repetitions	To enhance balance

**Table 3:** Minimum protection phase

Intervention	Dosage	Rationale
Shoulder, elbow, wrist, hip, and knee joint – active range of motion exercises	10 repetitions of a set	To maintain joint mobility
Hip-knee flexion in supine in air	A set of ten repetitions	To enhance the mobility of knee joint
Isometrics to shoulder, elbow, quadriceps, hamstrings, and vastus medialis oblique (VMO)	Ten repetitions of a set with fifteen seconds of hold	To strengthen the muscles
Static abdominals	Ten repetitions of a set with ten seconds of hold	To strengthen the muscles
Pelvic bridging	A set of ten repetitions with ten seconds of hold	To increase the hip extensor’s strength and stability of the trunk
Sit-to-stand activity	A set of ten repetitions	To make the patient stand
One leg stance with eyes closed with the support	Fifteen seconds hold with five repetitions	To enhance balance and static posture
Static quadriceps, hamstrings, and VMO in standing and dynamic quadriceps	10 repetitions with 10 seconds of hold	To strengthen the muscles
Toe standing and heel standing	A set of ten repetitions	To strengthen the muscles around ankle joint
Resistance band exercises to the shoulder, hip, knee, and ankle	A set of ten repetitions	To strengthen the muscles
Spot marching with support	For a minute	For weight bearing
Reach-outs in standing	Ten repetitions on both sides	To improve balance
Wall squats	Ten repetitions	To Strengthen
Gait training	Two laps	To improve gait

Concerning short-term goals, the rehabilitation programme is targeted at educating the patient, alleviating pain, enhancing joint functional ranges of motion, and initiating the strengthening of the muscles. Concerning long-term goals, it is intended at enhancing the full ranges of the joint, improving and maintaining the strength of the muscles, increasing endurance, and aiding the patient in resuming functional activity gradually.

In the maximum protection phase (Table 1), the joint mobility target improves and reduces pain. To alleviate the pain, interferential therapy (IFT) was applied for ten minutes for both knees. To enhance shoulder joint mobility, the shoulder pulley and shoulder wheel were for use. Heel slides improved the joint range of motion. To maintain the range of joints, active assisted range of motion exercises to the elbow, wrist, hip, and knee joints were given to the patient.

**Figure 4:**
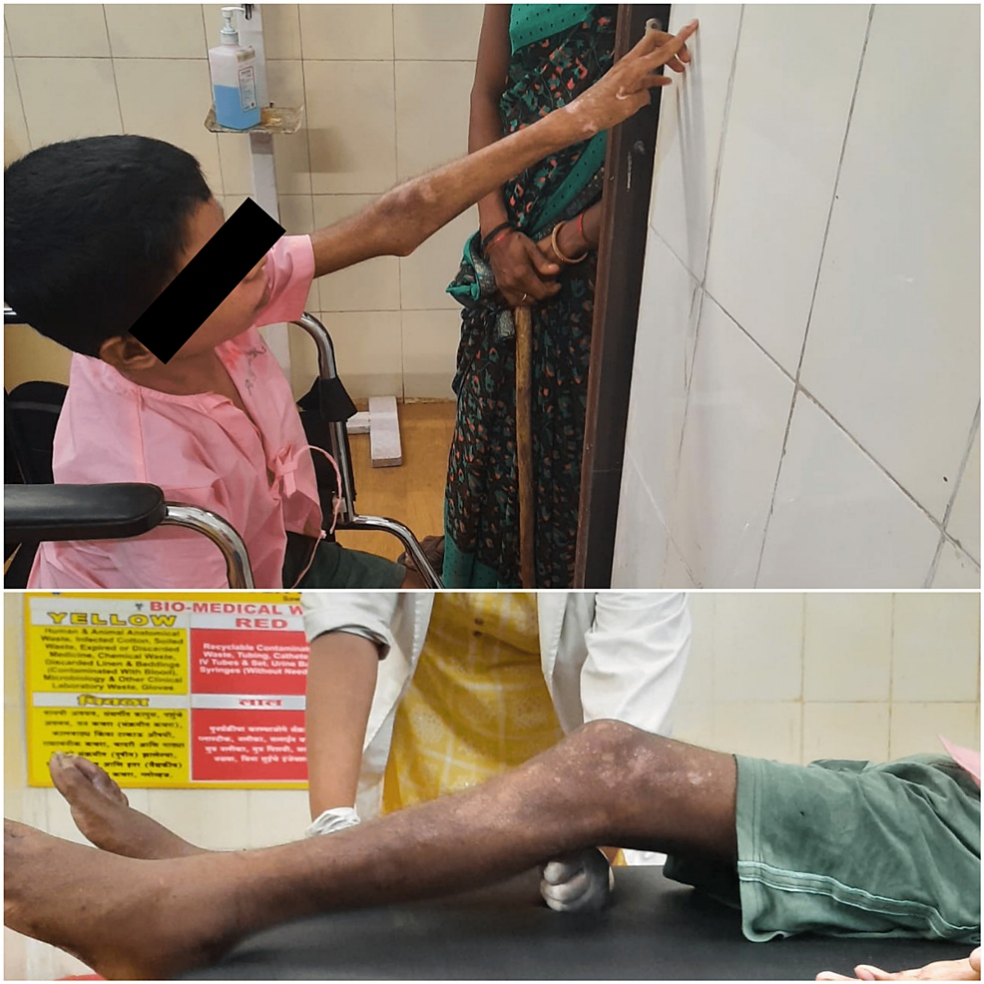
Patient during physiotherapeutic rehabilitation

In the moderate protection phase (Table 2), the target was to enhance joint mobility, build up strength, and initiate functional activity for the patient along with reduction of the pain. Mobility exercises like the active range of motion exercises to the shoulder, elbow, wrist, hip, and knee joint, and heel slides in supine were given (Figure 4). Isometric to the shoulder, elbow, quadriceps, hamstring, and vastus medialis oblique (VMO) along with cervical isometric exercises and static abdominal exercise were given in this phase as a strengthening regimen. Pelvic bridging was started, which is a weight-bearing exercise that enhances the hip extensor’s strength and promotes stability of the trunk. The sit-to-stand activity was begun for the patient.

In the minimum protection phase (Table 3), the target was to enhance joint mobility, increase strength, focus more on weight-bearing activities, and functional activity for the patient along with gait training. Mobility exercises were continued. Isometric to the shoulder, elbow, quadriceps, hamstring, and VMO along with cervical isometric exercises and static abdominal exercise were continued in this phase as a strengthening regimen. Pelvic bridging was started, which is a weight-bearing exercise that increases the hip extensor’s strength and promotes stability of the trunk. Static quadriceps, hamstrings, and VMO in standing and dynamic quadriceps were done to improve muscular strength. Resistance band exercises to the shoulder, hip, knee, and ankle were started. All this was done as a strengthening regimen. Sit-to-stand activity was continued. Spot marching for weight bearing and reach-outs in standing were started. He was given gait training.

## Discussion

Rickets is a condition that affects children's growth plates and is caused by low mineral ion concentrations, which impairs mineralization and endochondral ossification with the most common aetiology being deficiency of vitamin D [1]. Pityriasis rubra pilaris is a papulosquamous inflammatory dermatosis that is idiopathic. It is distinguished by palmoplantar keratoderma, islands of sparing, and hyperkeratotic follicular papules that coalesce into scaly orange-red plaques. This occurs in about one in every 50,000 patients in India. One in 500 new patients in the pediatric population has the dermatologic disease, which is a greater frequency than in the general population [7]. Several skin diseases could predispose to rickets [11].

In many nations, vitamin D insufficiency is still a major public health issue [5]. In the Indian subcontinent, it continues to be a major health problem [12]. Rickets in occurring association with skin diseases is rare [11]. There are various subtypes, including hypophosphatemic rickets (vitamin-D resistant rickets secondary to renal phosphate wasting), vitamin D-dependent rickets (defects in the metabolism of vitamin D), and nutritional rickets (due to deficiency of dietary of vitamin D, calcium, and/or phosphate) [13]. Radiographs, a physical examination, biochemical testing, and medical history all lead to a diagnosis of rickets.

There are a number of afflicted bodily systems, but physical examination and imaging analysis show that the musculoskeletal system is the most apparent [14]. Congenital and early rickets are frequently accompanied by hypocalcemic seizures. The rachitic rosary is a typical feature in newborns with congenital rickets. Sweating, hypotonia, craniotabes, irritability, enlarged wrists, occipital flattening, Harrison’s groove, and frequent respiratory tract infections are other features of rickets in infancy. Delay in walking, bowing of leg, gait abnormality, and dentition delay are frequent presenting symptoms in older infants. All the skeletal system problems and deformities occurring need to be managed by medical as well as physiotherapeutic rehabilitation. The independent return of the patient's functional efficiency can be helped by prompt treatment and rehabilitation.

In the case study, six weeks of physiotherapeutic rehabilitation was given. This significantly improved the patient's functional independence by reducing pain and improving joint range of motion, muscle strength, endurance, and gait. This case study shows the value of comprehensive physical therapy with a case of pityriasis rubra pilaris with rickets in a 15-year-old boy.

## Conclusions

After six weeks of rehabilitation, there was an improvement in the ranges of the joint, strength of muscle, gait, and functional independence significantly using physical therapy techniques. This case study shows the value of comprehensive physical therapy in a case of pityriasis rubra pilaris with rickets in a 15-year-old boy.
